# A recurrent de novo missense mutation in *COL1A1* causes osteogenesis imperfecta type II and preterm delivery in Normande cattle

**DOI:** 10.1186/s12711-024-00909-3

**Published:** 2024-05-21

**Authors:** Julien Corbeau, Cécile Grohs, Jeanlin Jourdain, Mekki Boussaha, Florian Besnard, Anne Barbat, Vincent Plassard, Julie Rivière, Christophe Hamelin, Jeremy Mortier, Didier Boichard, Raphaël Guatteo, Aurélien Capitan

**Affiliations:** 1 BioEpAR, INRAE, Oniris, CS, 40706 Nantes, France; 2 INRAE, AgroParisTech, GABI, Université Paris Saclay, 78350 Jouy-en-Josas, France; 3 ELIANCE, 75012 Paris, France; 4 IDELE, 75012 Paris, France; 5 Ecole Nationale Vétérinaire d’Alfort, Maisons-Alfort, France; 6 INRAE, AgroParisTech, MICALIS, Université Paris-Saclay, 78350 Jouy-en-Josas, France; 7 INNOVAL, 35538 Noyal Sur Vilaine, France

## Abstract

**Background:**

Nine male and eight female calves born to a Normande artificial insemination bull named “Ly” were referred to the French National Observatory of Bovine Abnormalities for multiple fractures, shortened gestation, and stillbirth or perinatal mortality.

**Results:**

Using Illumina BovineSNP50 array genotypes from affected calves and 84 half-sib controls, the associated locus was mapped to a 6.5-Mb interval on chromosome 19, assuming autosomal inheritance with germline mosaicism. Subsequent comparison of the whole-genome sequences of one case and 5116 control genomes, followed by genotyping in the affected pedigree, identified a de novo missense substitution within the NC1 domain of the *COL1A1* gene (Chr19 g.36,473,965G > A; p.D1412N) as unique candidate variant. Interestingly, the affected residue was completely conserved among 243 vertebrate orthologs, and the same substitution in humans has been reported to cause type II osteogenesis imperfecta (OI), a connective tissue disorder that is characterized primarily by bone deformity and fragility. Moreover, three *COL1A1* mutations have been described to cause the same syndrome in cattle. Necropsy, computed tomography, radiology, and histology confirmed the diagnosis of type II OI, further supporting the causality of this variant. In addition, a detailed analysis of gestation length and perinatal mortality in 1387 offspring of Ly and more than 160,000 progeny of 63 control bulls allowed us to statistically confirm in a large pedigree the association between type II OI and preterm delivery, which is probably due to premature rupture of fetal membranes and has been reported in several isolated cases of type II OI in humans and cattle. Finally, analysis of perinatal mortality rates and segregation distortion supported a low level of germ cell mosaicism in Ly, with an estimate of 4.5% to 7.7% of mutant sperm and thus 63 to 107 affected calves born. These numbers contrast with the 17 cases reported and raise concerns about the underreporting of congenital defects to heredo-surveillance platforms, even for textbook genetic syndromes.

**Conclusions:**

In conclusion, we describe a large animal model for a recurrent substitution in *COL1A1* that is responsible for type II OI in humans. More generally, this study highlights the utility of such datasets and large half-sib families available in livestock species to characterize sporadic genetic defects.

**Supplementary Information:**

The online version contains supplementary material available at 10.1186/s12711-024-00909-3.

## Background

In vertebrates, osteogenesis imperfecta (OI) is a group of at least 15 inherited disorders of connective tissue, most of which are autosomal dominant and characterized by bone deformity and fragility, with the possible addition of other pathological signs and varying degrees of severity [1–6]. Studies in humans have shown that mutations in just two genes encoding the alpha 1 and alpha 2 chains of collagen type I (*COL1A1* and *COL1A2*), the most abundant bone protein, account for 77% of cases, with the remainder involving at least 17 other genes (e.g., [3, 7]). To date only three independent cases of OI have been characterized at the molecular level in cattle, all caused by mutations in the *COL1A1* gene [8–11].

From 2017 to 2019, 17 stillborn calves born from the same Normande artificial insemination (AI) sire with pathological signs suggestive of OI and abnormally short gestation lengths were referred to the French National Observatory for Bovine Abnormalities (ONAB; doi.org/10.15454/BRKOV3; [12]). The purpose of this study was to characterize the phenotypic and genetic features of this new syndrome.

## Methods

### Animals

Among the progeny of a single bull (hereafter referred to as “Ly”), nine male and eight female stillborn calves affected by congenital bone fragility were observed in 14 purebred Normande herds over a 15-month period. Veterinarians and AI technicians collected ear biopsies and photographs for all of them. However, because dead animals are collected by rendering companies in less than 24 h, the body of only one affected female calf, born after 275 days, could be recovered for extensive pathological examination. The body of an unrelated calf that died of acute neonatal diarrhea at 1 week of age was also collected to serve as a control. In addition to the material collected for this study, DNA samples and single nucleotide polymorphism (SNP) array genotypes generated for routine genomic evaluation purposes (see below) were available for the sire “Ly”, six dams of affected calves, 84 unaffected progeny of Ly, and 31 of their dams.

### Pedigree analysis

Genealogical information based on breeders’ declarations was extracted from the French national bovine database for the 17 affected calves and 1167 of their paternal half siblings that survived at least 48 h. A search for common ancestry between Ly and the dams of the calves was performed using the anc_comm option of the pedig package [13]. Finally the average inbreeding rate between the case and control groups was compared using a Student’s t-test.

### DNA extraction

DNA was isolated from blood, semen, or tissue samples using the Puregene Tissue and Blood kit (Qiagen). DNA quality was checked by electrophoresis and quantified using a Nanodrop spectrophotometer (ThermoFischer Scientific).

### Analysis of illumina SNP array genotypes

The bull Ly, 101 of its progeny (17 cases and 84 controls) and 37 of their dams were genotyped over time with various SNP arrays (Illumina Bovine SNP50, EuroG10K and EuroGMD, San Diego, USA). Genotypes were subjected to parentage verification, phasing and imputation to the Bovine SNP50 using the FImpute3 software [14] as part of the French genomic evaluation, as described in Mesbah-Uddin et al*.* [15]. As a first step, we sought to determine whether the defect was due to an interchromosomal rearrangement and, thus, performed a genome-wide scan for abnormal linkage disequilibrium (LD) patterns between markers from nonhomologous chromosomes as described in Jourdain et al. [16]. Then, assuming dominant inheritance with germline mosaicism in the sire, the OI locus was mapped using transmission disequilibrium tests. For 15,195 informative markers (i.e., heterozygous in Ly), the proportion of each paternal allele transmitted to the case and control groups was compared using Fisher’s exact test. Finally, the negative log10 transformation of the p-values was used to visualize the data using the R software version 4.0.1 [17] and the ggplot2 package version 3.3.2 [18]. Due to the high LD between markers on the paternal phases, the tests were not independent. Therefore, to adjust the multiple testing thresholds, we assumed an average of one recombination per chromosome per meiosis and divided the p-values corresponding to 0.05 and 0.001 by a factor of 58 (i.e., 2 × 29 autosomes).

After the discovery of the causative mutation in the *COL1A1* gene, we used allele transmission rates for two flanking informative markers within Ly’s genotyped healthy progeny (n = 84) to estimate the proportion of mosaicism in Ly’s germ cells as described in Besnard et al*.* [19]. Briefly, we assumed that control calves carrying the at-risk haplotype had inherited the ancestral version of this haplotype (i.e., before the mutation event). Based on this assumption, we calculated the proportion of affected gametes as (nHb–nHa)/(2 × nHb), where nHa is the number of carriers of the at-risk haplotype among half-sib controls and nHb the number of carriers of the alternative paternal haplotype among half-sib controls.

### Analysis of whole-genome sequences

The genome of an OI-affected calf was sequenced at a 17.8 × coverage on a NovaSeq platform (Illumina; 2 × 150 bp mode) after library preparation using the NEXTflex PCR-Free DNA Sequencing Kit (Perkin Elmer Applied Biosystem). Reads were aligned to the ARS-UCD1.2 bovine genome assembly [20] and processed in accordance with the guidelines of the 1000 Bull Genomes Project [21] for the detection of SNPs and small insertion-deletions (InDels). Assuming that the causative mutation is dominant and had occurred de novo in the sire of the affected calf, we retained only the heterozygous variants found in the mapping interval (positions 35,459,825 and 41,972,985 bp on *Bos taurus* chromosome (BTA)19) that were never observed in 5116 unrelated control genomes from Run 9 of the 1000 Bull Genomes Project [21]. Filtered variants were then annotated using Variant Effect Predictor (Ensembl release 110; www.ensembl.org/Tools/VEP). In addition, we detected structural variants (SV) within the mapping interval using the Delly [22], Lumpy [23] and Pindel [24] software, and applied the same filters after comparison with SV catalogs of 200 control genomes reported in Boussaha et al*.*, [25] and Letaief et al*.*, [26].

### Genotyping of the *COL1A1* candidate variant

DNA samples from Ly (extracted from semen), three affected calves and three half-sib controls carrying the same paternal haplotype but in the non-mutated version, as well as their six dams were genotyped for the candidate missense variant g.36,473,965G > A on BTA19 by PCR followed by Sanger sequencing. A segment of 521 bp was amplified with primers GCCTCCCAGAACATCACCTA and CTTTTCGGGGGTTTCAGTTT and the Go-Taq Flexi DNA Polymerase (Promega) in a Mastercycler pro thermocycler (Eppendorf), according to the manufacturer’s instructions. PCR products were purified and bidirectionally sequenced by Eurofins Genomics (Ebersberg, Germany). Finally, Sanger sequences were analyzed using the NovoSNP software [27].

### Necropsy and pathological examination

A detailed pathological examination of the frozen bodies of the case and control calves was performed. Due to the multiple fractures observed during handling, an initial computed tomography (CT) scan was performed at the National Veterinary School of Alfort, followed by radiography of the forelimbs and hindquarters and a complete necropsy. Special attention was paid to the deformation of the skeleton, the strength of the different bones, the presence of local hemorrhages, the mobility of the joints, the strength and appearance of the teeth and the color of the sclera to allow comparison with previous studies [28, 29]. In addition, the right fore and hind limbs of each calf were collected, boiled, cleaned of residual soft tissue, and bleached with 5% hydrogen peroxide, prior to partial skeletal reconstruction to highlight the fractures, loss of bone substance and limb shortening in the case compared to the control calf. Gross clinical descriptions were available for 16 additional affected half-sibs.

### Histology

First incisors and samples from the distal diaphyseal region of the femur were fixed in 10% neutral buffered formalin for 24 h at 4 °C and then decalcified in a solution of 10% EDTA in 1 × phosphate buffered saline (PBS) (pH 8) for 4 h and 30 min at 50 °C in a KOS microwave tissue processor (Milestone). Tissues were then dehydrated in a graded ethanol series, cleared in xylene and embedded in paraffin. Microtome sections (5 µm, Leica RM2245) were mounted on adhesive slides (Klinipath- KP-PRINTER ADHESIVES), deparaffinized, and stained with hematoxylin and eosin (HE). Slides were scanned with the Pannoramic Scan 150 and analyzed with the CaseCenter 2.9 viewer (3D Histech).

### Analysis of gestation length and perinatal mortality

Information on the date of artificial insemination of their dams, date of birth, and survival within 48 h was obtained from the French national bovine database for 1387 progeny of Ly and 161,413 Normande calves born in the same period and sired by 63 control AI bulls born in the same year as Ly (mean = 2439 ± 1747 progeny per sire). Pairwise comparisons were then made using Student's t-tests between the mean gestation lengths of the four groups of calves that died perinatally or survived more than 48 h among the progeny of Ly and the control population. Mean gestation lengths of the control population were calculated using intrafamilial averages to account for differences in the size of paternal half-sib families. We did not consider cohort, herd, or calf sex effects because the sex ratio in each family was balanced and Ly’s offspring were born in a relatively short period of time (3 years) on a large number of farms (n = 716) with many control calves of different paternal origins available (median = 219 calves and 33 sires).

## Results and discussion

### Gross phenotypic description and inheritance analysis

**Fig. 1:**
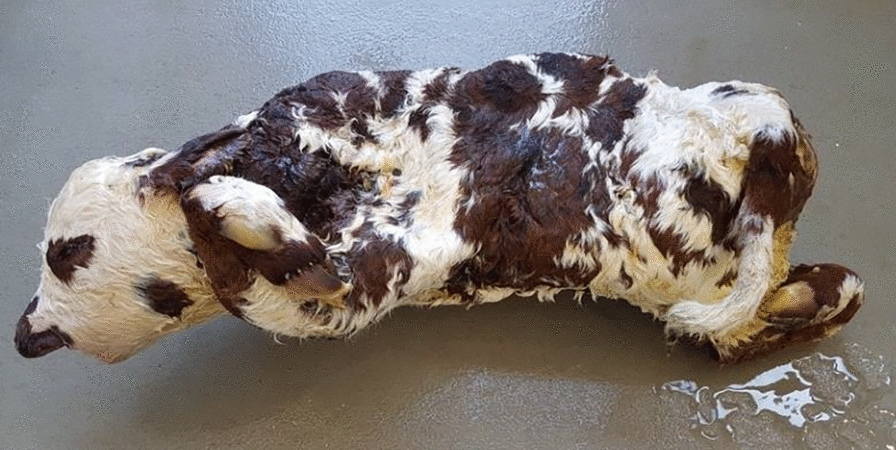
Picture of the female “corkscrew” calf necropsied in this study. Note the limb deformities that were systematically mentioned in the reports of this defect to the ONAB (see Additional file 1: Table S1), as well as the undershot jaw (brachygnathia inferior) observed in this particular individual

The nine male and eight female calves referred to the ONAB were born one to six weeks earlier than expected (see Additional file 1: Table S1). They were stillborn or died within minutes of birth from respiratory failure and had abnormally twisted and shortened limbs, earning them the nickname “corkscrew calves” (Fig. 1 and see Additional file 1: Table S1). Several breeders also reported fractures as a result of the dragging of dead calves by their limbs for transport. Pending further characterization (see below), these calves were classified as suffering from a congenital bone fragility syndrome, of which OI is the most common form. As mentioned in the Background section, the affected calves were registered on 14 different farms over a long period of time (15 months), and were all sired by the same bull (Ly), suggesting a genetic aetiology. According to the ONAB archives, in spite of thousands of reports of congenital malformations received over the past 20 years, no “corkscrew calves” have ever been reported outside of Ly’s progeny, and pedigree analysis did not reveal a significant increase in the inbreeding coefficient of the affected calves compared to 1167 of their paternal half siblings that survived at least 48 h, which did not support a recessive inheritance. Furthermore, Ly was found to be the worst Normande AI sire in terms of perinatal mortality with 15.9% of calves declared as dead within 48 h after birth compared to 8.2 ± 1.5% for 63 control sires used in the same period and born in the same year (Chi-square p-value = 4.3 × 10^−24^ considering 220 calves dead within 48 h and 1167 calves surviving longer for Ly versus 13,401 and 148,012 for the same categories, respectively, for the control population).

Considering all these elements and based on our experience (e.g., [16, 19]), we prioritized two possible modes of inheritance for this new genetic defect: Ly was a carrier of a de novo rearrangement involving two autosomes, or a de novo dominant mutation on one autosome, combined with germline and/or somatic mosaicism.

### Mapping and identification of a deleterious missense variant in the *COL1A1* gene

Under the first assumption, we analyzed LD between markers from nonhomologous chromosomes in 101 offspring of Ly (17 cases and 84 half-sib controls) with available phased and imputed Illumina Bovine SNP50 genotypes, as described in Jourdain et al. [16]. However, we did not find any abnormal LD patterns to support a possible interchromosomal rearrangement.

**Fig. 2:**
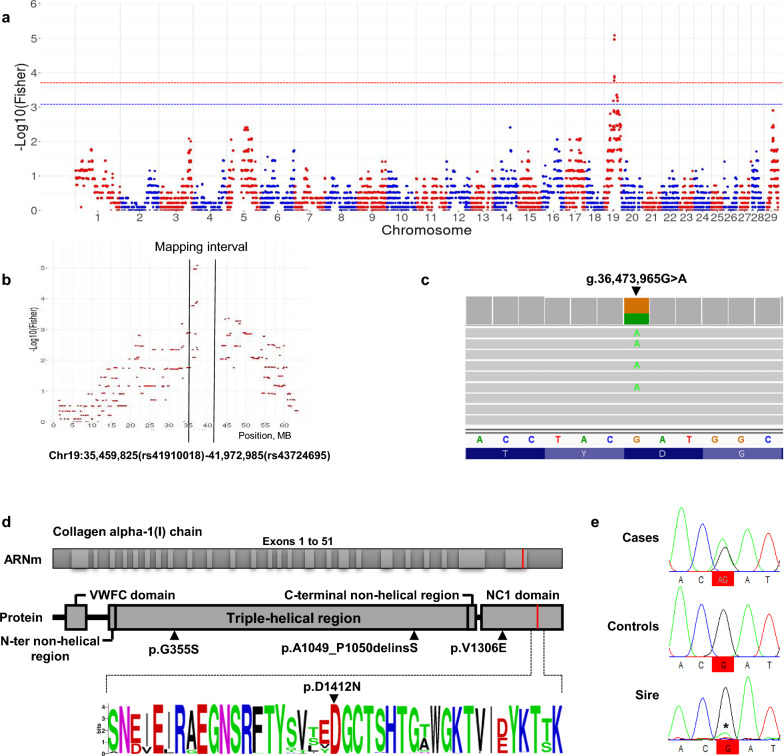
Mapping and identification of a de novo missense mutation in the *COL1A1* gene. **a**, **b** Manhattan plot of the results from transmission disequilibrium mapping, with a zoom on *Bos taurus* chromosome (BTA)19 (**b**). The dashed blue and red lines correspond to the 0.05 and 0.001 thresholds, respectively, after adjustment for multiple testing. **c** Integrative Genomic Viewer screenshot showing heterozygosity for a substitution at position g.36,473,965 of BTA19 in the whole-genome sequence of an affected calf. (**d**, top) Exon and domain information for the *COL1A1* gene and corresponding protein. The position of the p.D1412N substitution caused by the g.36,473,965G > A variant identified in this article and three previously described deleterious mutations in *COL1A1* in cattle are indicated on the protein (p.A1049_P1050delinsS in Bourneuf et al. [9]), p.G355S in Petersen et al*.* [10]), and p.V1306E in Jacinto et al*.*,[11]. (**d**, bottom) Logo representation of a multi-species alignment of 243 orthologs showing perfect conservation of the aspartic acid at position 1412 in cattle. Information on the protein ID in Ensembl and the amino acid sequences are provided in Additional file 3: Table S3. **e** Electropherograms of an affected calf, a control half-sib carrying the at-risk haplotype in its ancestral version (i.e., without the mutation), and their sire Ly for a segment of BTA19 encompassing the candidate variant. *Note the low proportion of allele *A* versus *G* in Ly supporting germline mosaicism. Electrophoregrams for a larger window are provided in Additional file 4: Figure S1

Under the second hypothesis, we applied transmission disequilibrium tests to the same dataset and mapped the corkscrew calf locus in a 8-Mb interval on BTA19 that includes 340 genes according to Ensembl release 110 (between positions 35,459,825 and 43,515,688 bp on the ARS-UCD1.2 bovine genome assembly; Fig. 2a and b).

We then sequenced the genome of an affected animal using Illumina technology, and identified 261 heterozygous candidate variants in the interval (231 SNPs, 28 short InDels, and 2 structural variants; (see Additional file 2: Table S2). By filtering out the variants observed in 5116 control genomes from more than 240 breeds, we reduced the list to a single candidate, namely a deleterious guanine to adenine substitution located at the end of the fiftieth and penultimate exon of the *COL1A1* gene (g.36,473,965G > A; Fig. 2c and d; SIFT score = 0).

This variant is predicted to cause the substitution of an aspartic acid residue, which is completely conserved among 243 vertebrate orthologs, by an asparagine within the NC1 domain of the COL1A1 protein (p.D1412N; Fig. 2d; see Additional file 3: Table S3). Interestingly, three independent mutation events causing the exact same p.D1412N substitution have been reported in human patients with the most severe form of OI, namely type II OI [30–32]. In addition, three mutations in *COL1A1* have been described to cause type II OI in cattle (p.G355S in Petersen et al*.* [10]; p.V1306E in Jacinto et al. [11]; and p.A1049_P1050delinsS in Bourneuf et al., [9]; Fig. 2d).

### Confirmation of the de novo nature and causality of the *COL1A1* missense mutation

For verification, we genotyped the g.36,473,965G > A variant using PCR and Sanger sequencing in the sire, three affected calves, three controls carrying the same paternal haplotype (i.e., predicted to carry the ancestral version of this at-risk haplotype without the mutation), and their six dams. The three cases were heterozygous whereas the controls and the dams were homozygous wild type. In addition, Sanger sequencing of the PCR performed on DNA extracted from Ly’s semen showed a small amount of the A allele (Fig. 2e; see Additional file 4: Figure S1). Taken together, these results support the de novo nature and the causality of this *COL1A1* missense mutation, as well as a low level of germline mosaicism in the sire.

### Detailed pathological examination of an affected calf support the diagnosis of type II OI

To better describe the phenotypic consequences of the *COL1A1* p.D1412N substitution in cattle and to confirm the putative diagnosis of type II OI, we performed a detailed pathological examination of an affected female calf that was frozen after death for preservation.

**Fig. 3:**
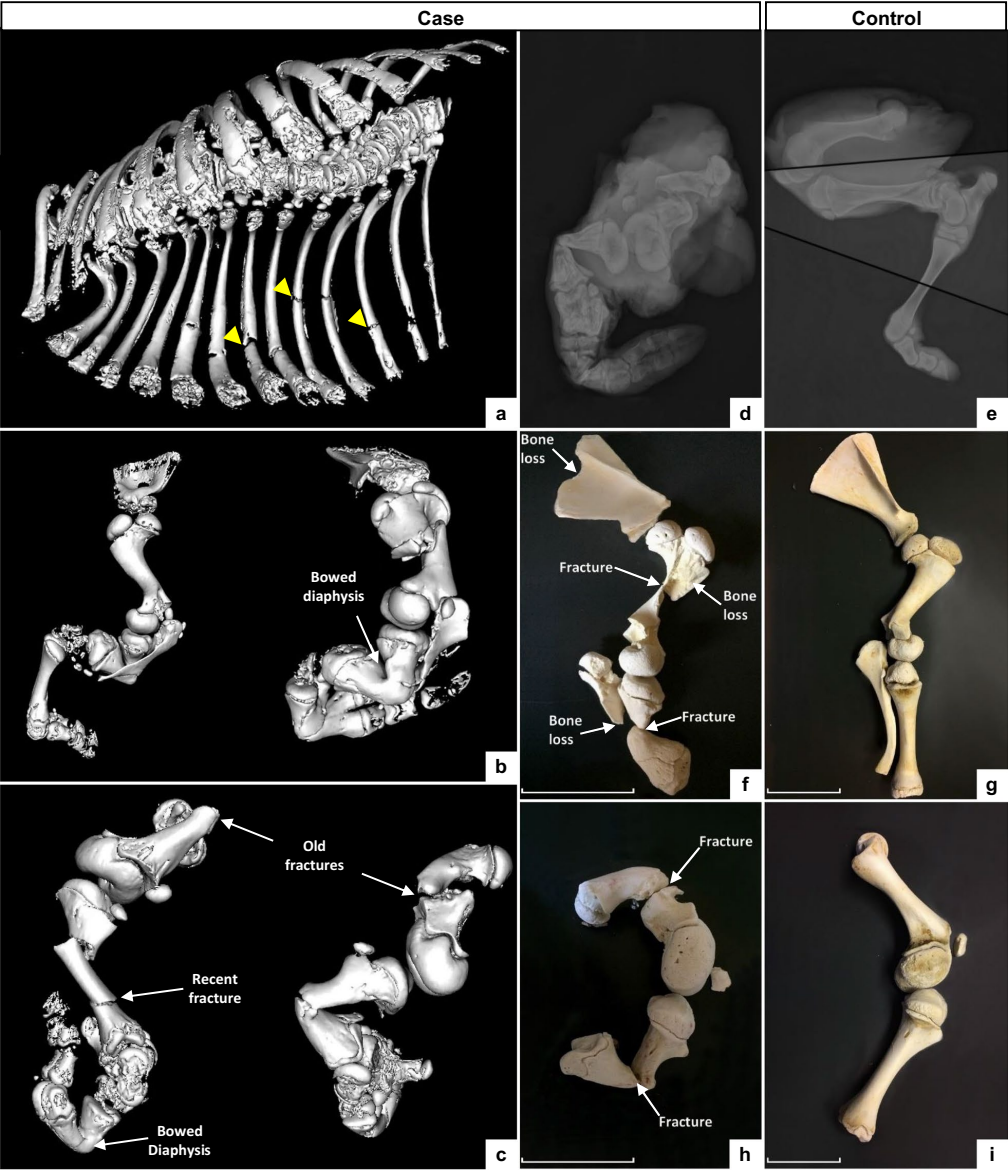
Computed tomography, radiography and partial skeletal reconstruction of a “corkscrew” calf and a matched control. **a**–**c** 3D reconstruction of CT images of the affected calf. Detail of the thoracic cavity (**a**), left thoracic limb in lateral view (**b**, left), right thoracic limb in medial view (**b**, right), left pelvic limb in lateral view (**c**, left) and right pelvic limb in medial view (**c**, right). Note the presence of multiple chronic and recent fractures and abnormal curvature of the diaphysis of several long bones. Yellow arrows highlight three of the multiple rib fractures seen in panel (**a**). **d**, **e** Radiographs of the posterior limbs of the affected (**d**) and control (**f**) calves. Fracture lines are highlighted. Note also the generalized decrease in bone opacity and cortical thinning, i.e., osteopenia, in the case compared to the control. **f**–**i** Partially reconstructed skeletons of the thoracic (upper panels) and pelvic (lower panels) limbs of the case (**f**, **h**) and control (**g**, **i**) calves, showing loss of bone substance, fractures, and bone remodeling in the affected calf. Scale bars correspond to 10 cm and their variation in size highlights the shortening of the limbs in the case compared to the control

A CT scan revealed multiple bone deformities consisting of bowed bones, chronic fractures associated with bone loss or abnormal healing, and numerous acute fractures of the limbs and ribs, likely due to the mechanical constraints of calving (Fig. 3a–c). This calf also suffered from generalized osteopenia, demonstrated by a diffuse decrease in opacity of the skeleton, as evidenced by a comparative radiographic analysis with a control calf (Fig. 3d and e).

At necropsy, the same affected calf presented with a 2-cm diameter umbilical hernia, light blue sclera, brachygnathia inferior and dentinogenesis imperfecta (Fig. 1 and see Additional file 5: Fig. S2, and not shown). The incisors were poorly erupted and showed a marked reduction in enamel thickness in spite of a normal external appearance (see Additional file 5: Fig. S2), while the premolars and molars were gray and brittle (not shown). Opening the chest cavity revealed multiple hematomas throughout the parietal layer of the pleura in addition to rib fractures and generalized atelectasis of all lung lobes, consistent with fetal death due to respiratory failure.

**Fig. 4:**
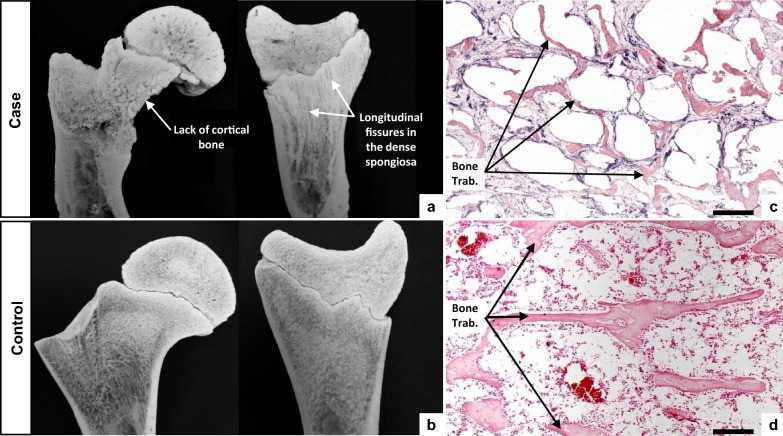
Macroscopic and microscopic structures of the bones of a “corkscrew” calf and a matched control. Macroscopic section of the proximal region of the femur (left panel) and distal region of the tibia (right panel) from the case (**a**) and control (**b**) calves. Note the absence of cortical bone and irregular spongiosa (i.e., cancellous bone). Histological sections of the femoral lamellar bone from affected (**c**) and control (**d**) calves stained with hematoxylin and eosin (HE). Scale bars in **c** and **d** correspond to 200 µm

Bone cleaning and partial skeletal reconstruction confirmed loss of bone substance and multiple fractures, and revealed arthrogryposis associated with capsule thickening in all limb joints except the coxofemoral joints (Fig. 3f–i). Longitudinal section of the long bones showed several macroscopic changes in their structure: the cancellous bone was very irregular and the cortical bone was thinner in all bones examined and even absent in the region of the proximal metaphysis of the femur (Fig. 4a and b). Histologic examination of the distal diaphyseal region of the humerus and femur also revealed a decrease in lamellar bone mass, with thinner and poorly mineralized trabeculae (Fig. 4c and d).

With the exception of the localized arthrogryposis, previously reported in a single calf with a de novo substitution in *COL1A1* (p.V1306E [11]), all of these pathologic features were consistent with classic observations in individuals with type II OI, who were heterozygous for the same p.D1412N substitution in the human orthologous protein or for other bovine *COL1A1* mutations [9–11, 30–32]. This led to the confirmation of the diagnosis of type II OI due to a dominant de novo missense mutation in *COL1A1*.

### Analysis of gestation length supports preterm birth in calves with type II OI

**Fig. 5:**
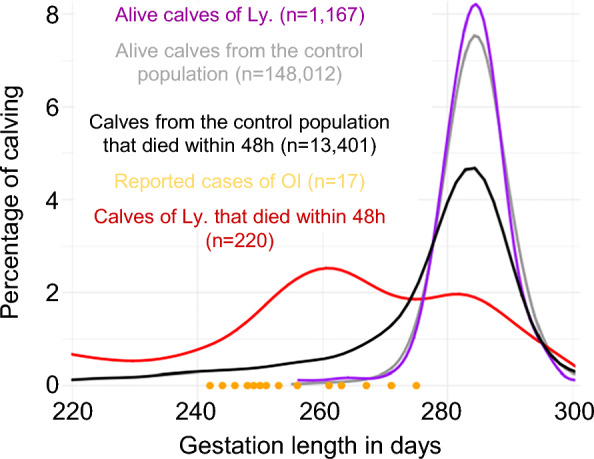
Analysis of the gestation length and perinatal mortality. Distribution of gestation lengths for the 17 reported cases of type II OI and for the calves that died or survived within 48 h of birth in the progeny of Ly or 63 control bulls

Since the breeders reported preterm birth, we examined gestation length and perinatal death in the offspring of Ly and 63 control bulls (Fig. 5). The gestation lengths of (i) the calves from Ly that survived more than 48 h; (ii) the calves from the control bulls that survived more than 48 h; and (iii) those from the control bulls that died within the first 48 h, all showed a nearly normal distribution with a maximum density of observations reached at around 283-284 days. In contrast, the distribution of the gestation lengths of the progeny of Ly that died perinatally was bimodal with a small peak approximately centered on the aforementioned peaks and a large peak centered around 261 days. Notably, the gestation lengths of the 17 cases fell within this larger peak, and their mean gestation length was reduced by 27.2 days, or 9.6%, compared with that of their paternal half-sibs that lived three days or more (mean and standard deviation were 256.1 ± 9.6 days and 283.3 ± 8.6 days for these two groups, respectively; Student t-test p-value = 3 × 10^−7^). Interestingly, several human epidemiologic studies have reported preterm delivery in isolated cases of neonates or stillborn fetuses diagnosed with type II OI (e.g., 19/21 cases in Sillence et al*.* [1], 2/3 cases in Cole et al*.* [33], and 4/8 cases in Rodriguez et al. [34]) and Jacinto et al*.* [11] also noted a shorter gestation (264 days) in an affected Holstein calf heterozygous for a *COL1A1* p.V1306E missense mutation. Here, the typical structure of the bovine population, consisting of large half-sib families that are spread over many farms, allowed us to statistically confirm the association between type II OI and preterm birth without possible bias introduced by confounding factors.

Since a recent study identified rare damaging missense variants in the *COL1A2*, *COL2A1*, and *COL5A1* genes associated with non-syndromic preterm premature rupture of membranes [35], and fibrillar collagens such as COL1A1 contribute to the tensile strength of fetal membranes [36, 37], we hypothesize that the preterm births observed in human and bovine cases of type II OI are due to the same phenomenon. Therefore, we strongly encourage colleagues to consider studying the fetal membranes of future cases of type II OI in vertebrates, which unfortunately was not possible in this case due to the timing of the sampling and subsequent phenotypic characterization.

### Analysis of perinatal mortality rates and segregation distortion supports a low level of germ cell mosaicism in Ly

Finally, we attempted to estimate the level of germline mosaicism in Ly using two approaches. On the one hand, we analyzed segregation distortion for two markers that are adjacent to the mutation in the 84 healthy control calves of Ly. We found 40 controls carrying the same paternal haplotype as the affected animals, but presumably in its ancestral version (i.e., without the de novo mutation), and 44 with the second paternal haplotype. From this 40:44 ratio, we estimated a rough proportion of 4.5% of mutant sperm ((44−40)/(2 × 44)). On the other hand, we looked at the perinatal mortality rate of Ly’s progeny. With 15.9% of calves declared as dead within 48 h of birth versus 8.2 ± 1.5% for 63 control sires, this bull was by far the worst Normande AI sire for this trait. Assuming that this excess of mortality was due to type II OI, we estimated the proportion of affected calves and mutant sperm to be approximately 7.7% (i.e., 15.9–8.2%). Using one or the other of these estimates, this would represent between 63 and 107 cases out of 1387 offspring of Ly, which contrasts with the 17 cases reported to us and raises concerns about the underreporting of congenital defects to heredo-surveillance platforms, even for textbook genetic syndromes.

## Conclusions

In conclusion, we describe a large animal model for a recurrent substitution in the *COL1A1* gene that is known to cause type II OI in humans, and statistically confirm the association of this syndrome with preterm birth, possibly due to premature rupture of membranes. More generally, this study highlights the usefulness of large datasets and large half-sib families available in livestock species to characterize genetic defects.

### Supplementary Information

**Additional file 1: Table S1.** Information on the Normande "corkscrew" calves sired by Ly and reported to the ONAB. AI: Artificial insemination. "Days preterm" refers to how early calves were born compared to the breed average gestation length of 284 days. The necropsied case is highlighted in bold.

**Additional file 2: Table S2.** List of heterozygous positional candidate variants found in the genome of a “corkscrew” calf. Structural variants refer to InDels larger than 50 bp detected by at least two different tools (see Methods). “Present_in_controls” indicates whether the variant was observed in at least one of the 5116 genomes from more than 240 breeds used as controls.

**Additional file 3: Table S3.** Extract from 243 vertebrate orthologs around the bovine COL1A1 p.D1412N substitution ± 20 amino acids. The complete protein sequences were obtained from Ensembl release 110 (www.ensembl.org/) and aligned using CLUSTALW (https://www.genome.jp/tools-bin/clustalw).

**Additional file 4: Figure S1.** Electropherograms of selected individuals for a 40-bp segment encompassing the *COL1A1* candidate variant. Electropherograms of an affected calf, a control half-sib carrying the at-risk haplotype in its ancestral version (i.e., without the mutation), and their sire Ly. The region displayed ranges from positions 36,473,946 to 36,473,985 bp on BTA19. The position of the candidate substitution is highlighted in red; *Note the low proportion of allele *A* versus *G* in Ly supporting germline mosaicism.

**Additional file 5: Figure S2.** Macroscopic and microscopic view of the teeth of case and control calves. (**a**, **b**) Images of the lower jaw showing poorly erupted incisors in the affected calf (**a**) compared to the control (**b**). (**c**, **d**) Histological sections of the first incisor stained with HE. De: Dentin. Dp: Dental pulp. Note the marked reduction in dentin thickness and the irregular shape of the margin between the dentin and pulp cavity in the case (**c**) compared to the control calf (**d**). Both are features of OI.

## Data Availability

The WGS data of the OI-affected calf are available at the European Nucleotide Archive (www.ebi.ac.uk/ena) under the study accession no. (Project PRJEB64022; Sample ERS16092187). The laboratory protocols for DNA isolation, genotyping of candidate variants, histology and necropsy analyses have been deposited on the protocols.io platform and are available under the following Doi: 10.17504/protocols.io.kqdg3xojeg25/v1, 10.17504/protocols.io.rm7vzxp32gx1/v1, 10.17504/protocols.io.4r3l22wn3l1y/v1, and 10.17504/protocols.io.n2bvj344wlk5/v1, respectively.
