# Supplementary figures and images for: A recurrent de novo missense mutation in COL1A1 causes osteogenesis imperfecta type II and preterm delivery in Normande cattle
Source: Genet Sel Evol. 2024 May 21;56:39. doi: 10.1186/s12711-024-00909-3 (PMC11107018; doi:10.1186/s12711-024-00909-3)

**Expected traces  
based on ARS-  
UCD1.2  
assembly**

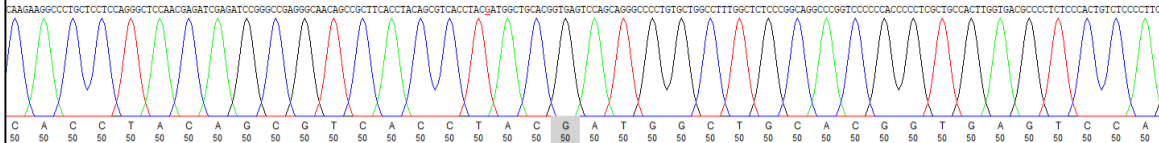

**Case**

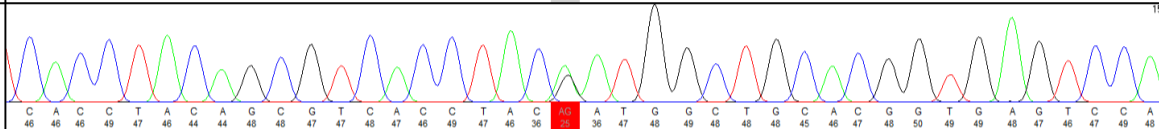

**Control**

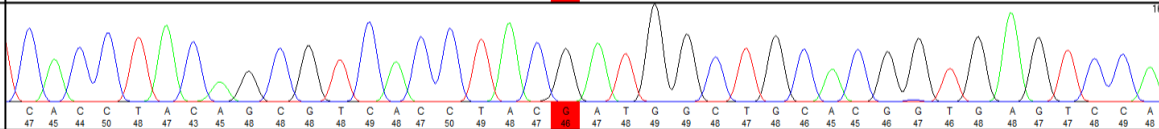

**Sire**

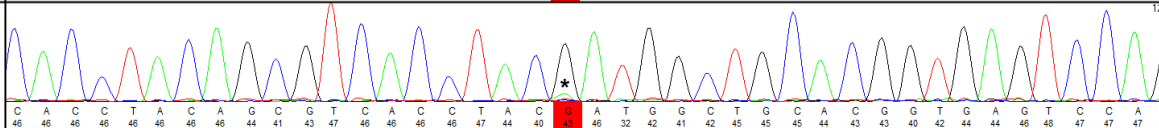

Supplement: Supplementary file 1 — Additional file 1: Table S1. Information on the Normande "corkscrew" calves sired by Ly and reported to the ONAB. AI: Artificial insemination. "Days preterm" refers to how early calves were born compared to the breed average gestation length of 284 days. The necropsied case is highlighted in bold. [file 12711_2024_909_MOESM1_ESM.pdf]

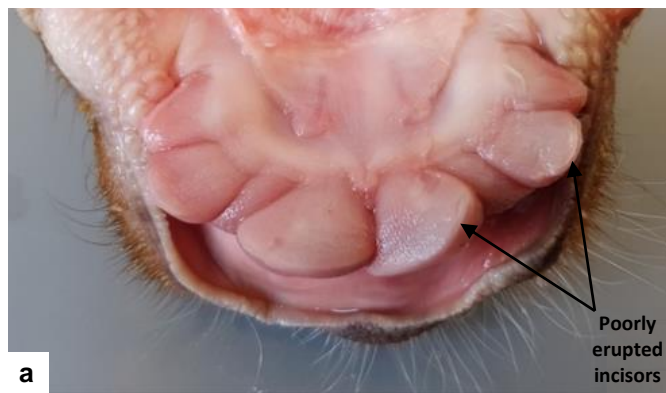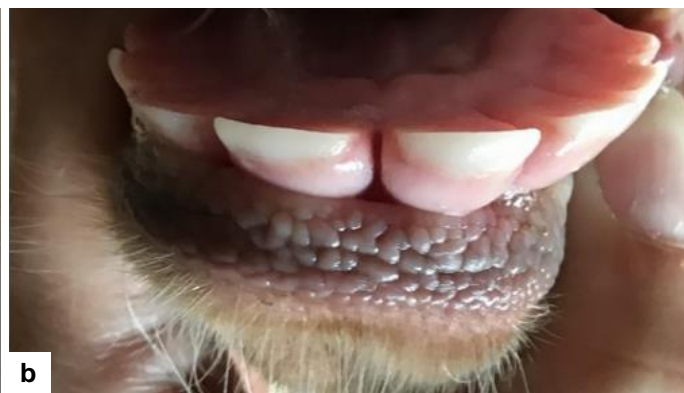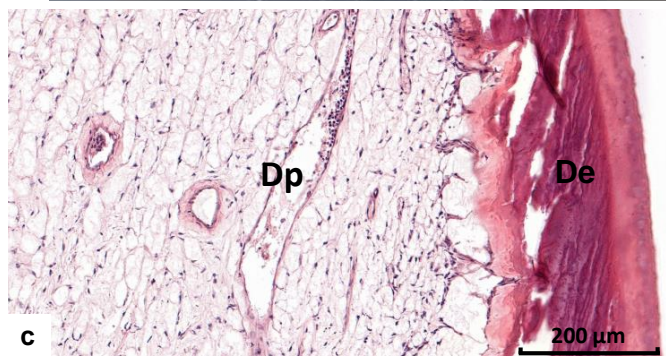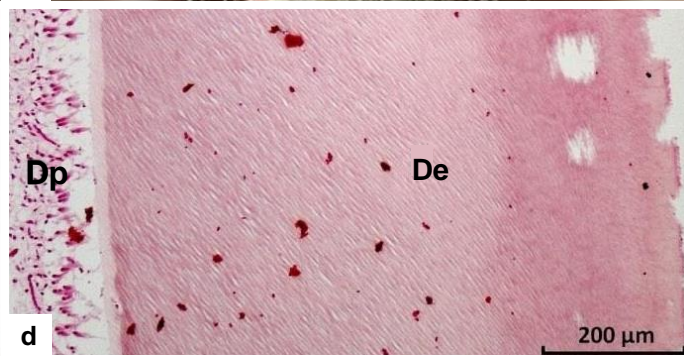

Supplement: Supplementary file 2 — Additional file 2: Table S2. List of heterozygous positional candidate variants found in the genome of a “corkscrew” calf. Structural variants refer to InDels larger than 50 bp detected by at least two different tools (see Methods). “Present_in_controls” indicates whether the variant was observed in at least one of the 5116 genomes from more than 240 breeds used as controls. [file 12711_2024_909_MOESM2_ESM.pdf]
